# Lipoteichoic Acid Stimulation of Macrophages Causes Mitochondrial Dysfunction

**DOI:** 10.1016/j.identj.2025.109290

**Published:** 2025-12-19

**Authors:** Siyao Liu, Xin Liu, Guixin Li, Huan Liu, Weiwei Zhang, Shuang Pan

**Affiliations:** a The First Affiliated Hospital of Harbin Medical University and Department of Endodontics, School of Stomatology, Harbin Medical University, Harbin, P. R. China; b Department of Stomatology, Linyi People's Hospital, Linyi, P. R. China; c Department of Stomatology, Second Affiliated Hospital of Harbin Medical University, Harbin, P. R. China

**Keywords:** Macrophage, Apical periodontitis, Mitochondrial, Lipoteichoic acid

## Abstract

**Objectives:**

This study aimed to investigate the role of mitochondrial dysfunction in the pathogenesis of *Enterococcus faecalis* and its lipoteichoic acid (LTA)-induced refractory apical periodontitis, and then to evaluate whether the modulation of mitochondrial dynamics with Mdivi-1 could alleviate the ensuing inflammatory response..

**Methods:**

An LTA-induced macrophage model was established to simulate the inflammatory environment of apical periodontitis. Changes in inflammatory factors, mitochondrial morphology, dynamics-related proteins, autophagy markers, and reactive oxygen species (ROS) were analysed. The mitochondrial division inhibitor Mdivi-1 was applied to assess its effects on mitochondrial and inflammatory parameters.

**Results:**

In the *in vivo* model, *E. faecalis* infection successfully induced apical periodontitis, as confirmed by radiographic evidence of periapical bone loss and histological observation of inflammatory cell infiltration. These lesions exhibited a significant upregulation of the pro-inflammatory marker iNOS, concurrently with a downregulation of the mitochondrial protein MFN-2. Consistent with the *in vivo* findings, LTA stimulation in a cellular model significantly increased the expression of inflammatory mediators (NLRC4, iNOS, NF-κB, Caspase-1) and induced mitochondrial dysfunction, characterised by morphological disruption, dysregulated dynamics, impaired autophagy and elevated ROS levels. Critically, Mdivi-1 treatment mitigated these abnormalities by improving mitochondrial structure and function, normalising dynamics-related protein expression and consequently reducing the inflammatory response.

**Conclusions:**

Mitochondrial dysfunction plays a central role in LTA-driven inflammatory processes in apical periodontitis. Targeting mitochondrial dynamics with Mdivi-1 can restore mitochondrial function and mitigate macrophage-mediated inflammation, revealing a key mechanism underlying refractory apical periodontitis.

**Clinical Significance:**

This study suggests that enhancing mitochondrial function with agents such as Mdivi-1 could serve as a novel therapeutic strategy for refractory apical periodontitis by modulating immune responses and reducing chronic inflammation, potentially improving treatment outcomes in clinically challenging cases.

## Introduction

Refractory apical periodontitis (RAP) is a persistent inflammatory disease characterised by progressive alveolar bone destruction that persists after standard root canal treatment.^1^ *Enterococcus faecalis* is established as the primary pathogen in RAP because of its unique ecological advantages in post-treatment root canals.^2^ Clinical studies consistently identify *E. faecalis* as the most prevalent species in refractory cases, where its ability to invade dentinal tubules, form resilient biofilms and thrive under nutrient stress enables survival in the treated root canal environment.^3^^,^^4^ The pathogenicity of *E. faecalis* in RAP is largely mediated through its interaction with macrophages, the key immune regulators in periapical lesions.^5^ The major virulence factor lipoteichoic acid (LTA) from *E. faecalis* triggers significant pro-inflammatory responses in macrophages, including the release of TNF-α and nitric oxide.^6^ Furthermore, LTA activates the NLRP3 inflammasome through NF-κB–dependent pathways and promotes macrophage polarization toward the pro-inflammatory M1 phenotype, establishing a direct mechanism by which *E. faecalis* maintains the chronic inflammatory state and bone destruction characteristic of RAP.^7^

Mitochondria are increasingly recognised as central signalling hubs in macrophage immunoregulation.^8^ Disruptions in mitochondrial dynamics and alterations in mitochondrial function are essential for macrophage activation.^9^ As highly dynamic organelles, mitochondria maintain their equilibrium through fusion, fission, and cristae reshaping, which are critical to sustaining normal physiological functions.^10^ Increasing evidence underlines the crucial function of mitochondria as central intracellular signalling platforms that regulate innate immunity and inflammatory processes.^11^^,^^12^ Specifically, pro-inflammatory differentiated macrophages transition their primary glucose metabolism from mitochondrial oxidative phosphorylation to aerobic glycolysis.^13^ Consequently, inhibiting glycolysis reduces LPS-induced ROS and pro-inflammatory factor production.^14^ These dynamic organelles influence inflammatory responses through a fusion–fission balance, metabolic reprogramming and the release of mtROS and mtDNA.

Mdivi-1, identified through chemical screening using the model organism *Saccharomyces cerevisiae*, has been shown to inhibit mitochondrial division in both yeast and mammals.^15^ Under experimental conditions in brewing yeast, mdivi-1 impedes mitochondrial division without significantly affecting cellular growth.^16^ The presumed target of mdivi-1 is the GTPase Dnm1, which is essential for mitochondrial division.^17^ Considered a potential therapeutic agent, mdivi-1 is being explored for its applications in treating stroke,^18^ cardiovascular diseases^19^^,^^20^ and neurodegenerative disorders.^21^, ^22^, ^23^ Additionally, it has been reported to enhance immune-mediated tumour control.^24^^,^^25^ While the mitochondrial fission inhibitor Mdivi-1 has demonstrated therapeutic potential in neurovascular and immune-related disorders, its role in modulating macrophage-driven inflammation in RAP remains unexplored.

Currently, the relationship between mitochondrial dysfunction and LTA-induced macrophage inflammation in RAP is poorly defined. It is unknown whether LTA disrupts mitochondrial dynamics and function in macrophages, and if so, whether pharmacological inhibition of mitochondrial fission can attenuate such damage and subsequent inflammation. This study therefore aimed to investigate mitochondrial morphological and functional alterations in LTA-stimulated macrophages and evaluate the therapeutic potential of Mdivi-1. Using transmission electron microscopy, confocal imaging, and Western blotting, we assessed mitochondrial integrity, dynamics-related protein expression, and inflammatory activation. Our findings demonstrate that Mdivi-1 restores mitochondrial homeostasis and mitigates inflammatory responses, suggesting mitochondrial targeting as a promising strategy for managing refractory apical periodontitis.

## Materials and methods

### Cell culture and disposal

RAW264.7 (mouse mononuclear macrophage leukaemia cells), kindly provided by the Second Affiliated Hospital of Harbin Medical University, were cultured in Dulbecco’s modified Eagle’s medium enriched with 10% foetal bovine serum. Cells were incubated at 37 °C with 5% CO_2_ in a cell culture incubator for subsequent experiments. An optimal concentration of LTA that does not impair cell growth was identified to establish an inflammation model in macrophages. Furthermore, the suitable concentration of Mdivi-1 was determined, and RAW264.7 cells were pretreated for 30 minutes with Mdivi-1 prior to the addition of LTA to develop the treatment group model.

### HE staining

Following the respective treatments with LTA and Mdivi-1, cells were fixed and subjected to standard haematoxylin and eosin (H&E) staining. Cells were stained with haematoxylin to visualise nuclei and subsequently with eosin to counterstain cytoplasmic and extracellular components. Cells from all groups (control, inflammation and treatment) were then observed and imaged under a microscope using consistent fields of view. This protocol allowed for a clear visual assessment of morphological changes and aided in determining the optimal treatment duration for LTA and Mdivi-1.

### Western blot

Cellular proteins were extracted and quantified using a bicinchoninic acid (BCA) assay kit. Experimental groups were established, and proteins were subsequently transferred to polyvinylidene fluoride (PVDF) membranes via electrophoresis and blotting. Following blocking with 5% non-fat milk, the membranes were incubated overnight at 4 °C with the following primary antibodies: rabbit anti-NLRC4 (1:1000, #25719), rabbit anti-NF-B (1:1000, #ab178945), rabbit anti-NF-κB (1:1000, #8242), rabbit anti-Caspase-1 (1:1000, #ab179515), rabbit anti-Drp1 (1:1000, #8570), rabbit anti-p-Drp1 (ser637) (1:1000, #6319), rabbit anti-MFN-2 (1:1000, #9842), mouse anti-Parkin (1:1000, ab77924), rabbit anti-HIF-1α (1:1000, #ab179483), rabbit anti-MT-CO2 (1:1000, #ab79393), rabbit anti-MT-CYB (1:1000, #ab198860) and rabbit anti-MT-ND1 (1:1000, #ab181848). This was followed by incubation with species-matched secondary antibodies: anti-rabbit IgG (1:2000, #7074) and anti-mouse IgG (1:2000, #7076). β-actin (1:1000, #4970) and Vinculin (1:1000, #13901) served as internal controls. Protein bands were visualised using enhanced chemiluminescence (ECL) substrate and analysed using ImageJ software.

### Transmission electron microscopy

Various cell groups were collected and fixed in 2.5% glutaraldehyde at 4 °C for 24 hours, followed by fixation in 1% osmium tetroxide for 2 hours. Subsequently, the samples were dehydrated in graded ethanol and embed in epoxy resin. Mitochondrial morphology was observed using a transmission electron microscope (H7650).

### Confocal laser scanning microscopy

Cells were cultured in a 6-well plate containing cover glass at a density of 1 × 10^5^ cells per well. After washing with PBS, the cells were incubated with MitoTracker Red CMXRos at 37 °C for 30 minutes. Subsequently, the cells were fixed with 4% paraformaldehyde, and the nuclei were stained with DAPI-containing antifade mounting medium. After drying, mitochondrial morphology was assessed using a confocal laser scanning microscope (model LSM 880). Intracellular reactive oxygen species levels were detected using BODIPY 581/591 C11 lipid peroxidation sensor, and the green fluorescence intensity was analysed using CLSM. All quantitative image data were processed using ImageJ software.

### Establishment of the animal model

Ten female Sprague-Dawley rats, aged 2 months and weighing approximately 180 g each, were used in this study. General anaesthesia was induced by intraperitoneal injection of a 10% chloral hydrate solution (1 mL/kg body weight). The absence of a blood draw upon needle aspiration confirmed correct intraperitoneal placement, and successful anaesthesia was determined by the loss of response to external stimuli and the maintenance of regular cardiorespiratory function. Following anaesthesia, the mandibular left first molar was surgically exposed. The pulp chamber was accessed using a high-speed handpiece with a 1/4 round bur under continuous saline irrigation to prevent thermal damage. Subsequently, 5 µL of an *E. faecalis* suspension (1.0 × 10^8^ CFU/mL) was carefully inoculated into the exposed pulp cavity using a microsyringe. The access cavity was then sealed with Fuji IX glass ionomer cement to establish a contained infection. The contralateral mandibular first molar received no intervention and served as the internal negative control. All experimental procedures were approved by the Institutional Animal Care and Use Committee (IACUC) of the first affiliated hospital of Harbin Medical University (2024030).

### Histological processing, staining and imaging

Following a 4-week experimental period, rats were anaesthetized with 10% chloral hydrate and perfused transcardially with 4% paraformaldehyde (PFA) in 0.1 M phosphate buffer (pH 7.4). Mandibles were dissected and post-fixed in the same fixative for 48 hours at 4 °C. After removal of adherent soft tissues, samples were decalcified in 15% EDTA (pH 7.4) for 4 weeks. The decalcified tissues were rinsed, dehydrated through a graded ethanol series, cleared in xylene and embedded in paraffin. Serial sections of 4-μm thickness were cut and mounted on glass slides.

Isolated mandibles were imaged using a high-resolution X-ray system at 20 kV with a 300-second exposure. Image contrast was optimised using the accompanying FAXitron DX software (v2.0).

Deparaffinised sections were stained with hematoxylin, differentiated in 1% acid alcohol, blued in running tap water, counterstained with eosin, dehydrated, cleared and mounted with neutral balsam.

Antigen retrieval was performed by heating sections in citrate buffer (pH 6.0) under pressure. Endogenous peroxidase activity was quenched using 3% H_2_O_2_. Sections were blocked with 3% bovine serum albumin and incubated overnight at 4 °C with primary antibodies against iNOS (1:500), MFN-2 (1:50) and Parkin (1:50). After washing, sections were incubated with an HRP-conjugated secondary antibody, and the signal was developed using 3,3′-diaminobenzidine (DAB). Counterstaining was performed with haematoxylin. All stained sections were examined and imaged under a light microscope.

### Statistical analysis

Statistical analysis was conducted using SPSS 17.0 software. Data are presented as the mean ± standard deviation of 3 independent experiments. Two-group comparisons were performed using *t*-tests. One-way analysis of variance (ANOVA) was employed, followed by *post hoc* tests (LSD) using the least significant difference method for multiple comparisons. Differences were deemed statistically significant if the 2-tailed probability (*P*) value was less than .05.

## Results

### Infection with E. faecalis induces periapical periodontitis in rats

**Fig. 1:**
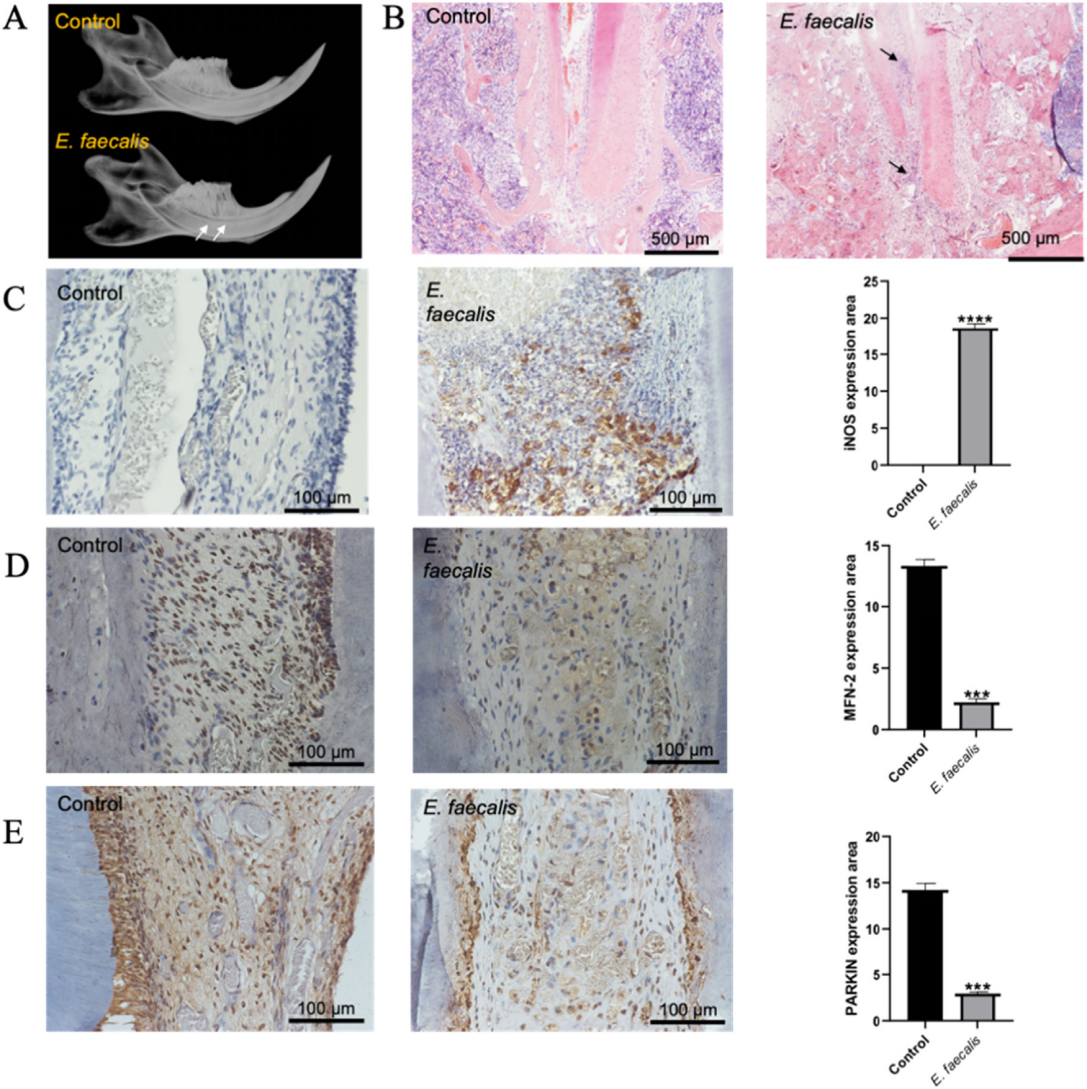
*E. faecalis* infection induces apical periodontitis associated with inflammatory activation and mitochondrial dysregulation in a rat model. A, Representative radiographic images of the mandibular first molar. The white arrow indicates a periapical radiolucent area, signifying bone loss due to infection. B, Haematoxylin and eosin (H&E) staining of the periapical region. The black arrow denotes a dense aggregation of inflammatory cells. C, Immunohistochemical (IHC) analysis and quantitative analysis of iNOS expression in the periapical lesion (****, *P* < .0001). D, IHC staining and quantitative analysis of MFN-2 expression (***, *P* < .001). E, IHC staining and quantitative analysis of Parkin expression (***, *P* < .001).

Refractory apical periodontitis (RAP), predominantly caused by *Enterococcus faecalis*, presents significant therapeutic challenges in clinical practice. A key virulence factor of this pathogen, lipoteichoic acid (LTA), is known to induce TNF-α and NF-κB expression in macrophages, thereby exacerbating inflammatory responses. In this study, a solution containing cultured *E. faecalis* was injected into the pulp cavity of the first mandibular molar in rats, which were then observed over a 4-week feeding period before being euthanised. Subsequently, the mandibles were extracted, and X-rays were used to examine the apical periodontium of the molar. As illustrated in Figure 1A, compared with controls, the experimental group displayed defects in the hard tissues of the molar crown, with low-density lesions evident around the apex. Horizontal resorption of the alveolar bone at the tooth's neck was also noted, signifying an inflammatory response in the apical periodontium induced by *E. faecalis*. As depicted in Figure 1B, H&E staining showed dense blue-stained cell nuclei concentrated in the apical third and near the apical foramen in the experimental group, indicating concentrated inflammatory cells in the pulp and periapical tissues post–*E. faecalis* infection. Further, as shown in Figure 1C, analysis of iNOS protein expression revealed significantly higher levels in the experimental group (*P* < .001), suggesting the involvement of pro-inflammatory M1 macrophages in the progression of *E. faecalis*–induced apical periodontitis. Figure 1D and 1E shows reduced expression of MFN-2 and Parkin in the pulp of the experimental group compared to controls (*P* < .001), suggesting that *E. faecalis* infection may alter mitochondrial fusion, autophagy levels and mitochondrial physiological activity, potentially playing a role in the disease's progression. However, the specific regulatory mechanisms of macrophages in *E. faecalis*–induced apical periodontitis through mitochondrial pathways require further investigation using cellular models.

### Stimulation with LTA induces an inflammatory response in macrophages

**Fig. 2:**
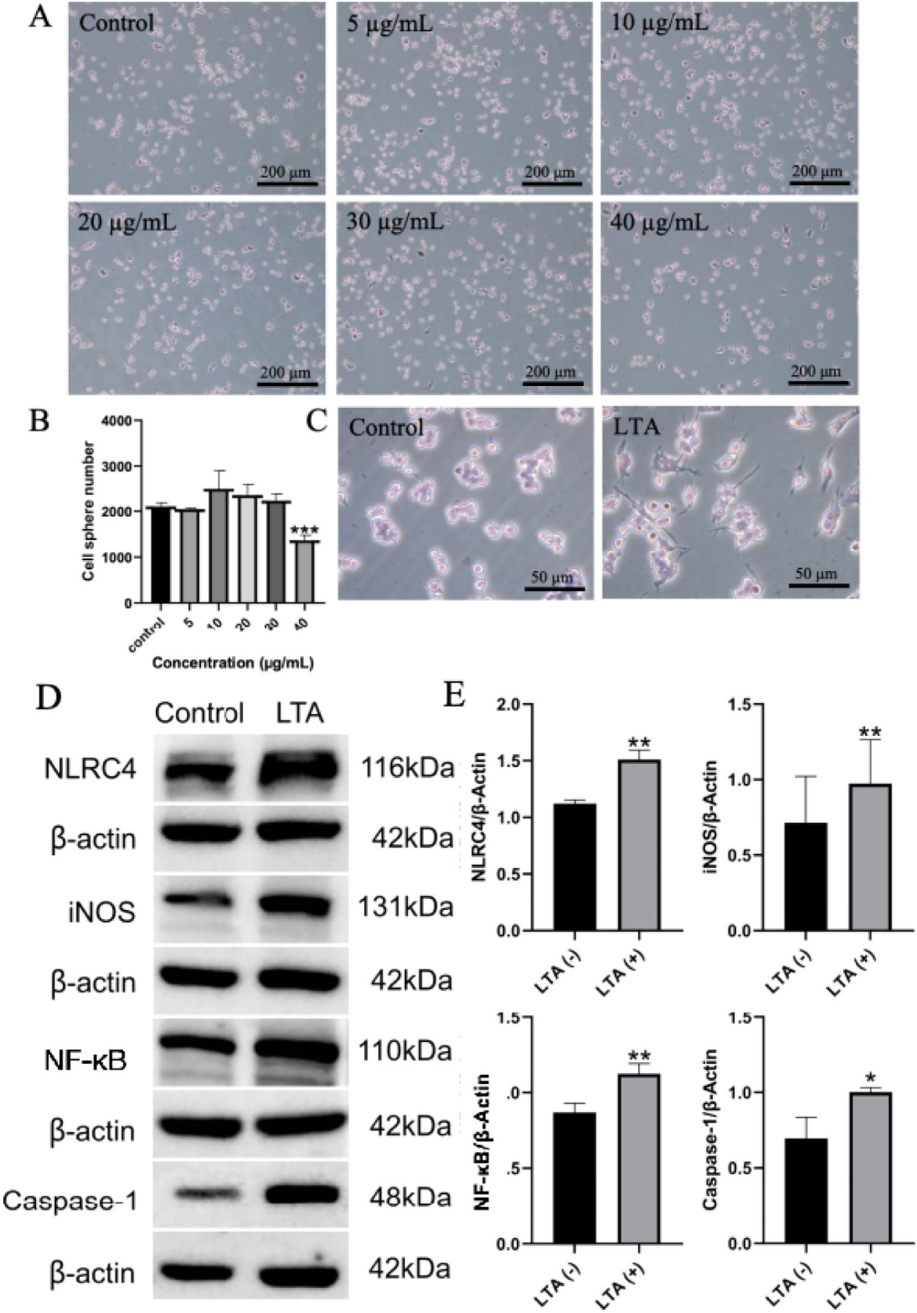
Inflammatory response of macrophages induced by lipoteichoic acid stimulation. A and B, The effect and quantitative analysis of different concentrations of lipoteichoic acid on the number of macrophages (***, *P* <.001). C, 30 µg/mL lipoteichoic acid stimulated macrophages for 36 hours (H&E staining). D and E, Immunoblot analysis and quantitative analysis of inflammatory factors in RAW264.7 cells stimulated by lipoteichoic acid for 36 hours (*, *P* < .05; **, *P* < .01).

LTA, a key virulence factor of *E. faecalis*, triggers a cascade of inflammatory responses in macrophages. Haematoxylin and eosin staining showed that a 24-hour exposure to 40 µg/mL LTA significantly reduced macrophage numbers (Figure 2A, B). Based on this observation, 30 µg/mL LTA was selected to establish an inflammatory model. Following 36 hours of exposure at this concentration, macrophages developed numerous pseudopods and accumulated substantial vesicular material within the cytoplasm, suggesting enhanced phagocytic activity (Figure 2C). Western blot analysis demonstrated that the expression levels of NLR family CARD domain-containing protein 4 (NLRC4), M1 pro-inflammatory marker iNOS, inflammatory mediator NF-κB and apoptotic protein Caspase-1 were significantly elevated in the infected group compared to the control group (Figure 2D, E).

### LTA stimulation induces alterations in mitochondrial morphology

**Fig. 3:**
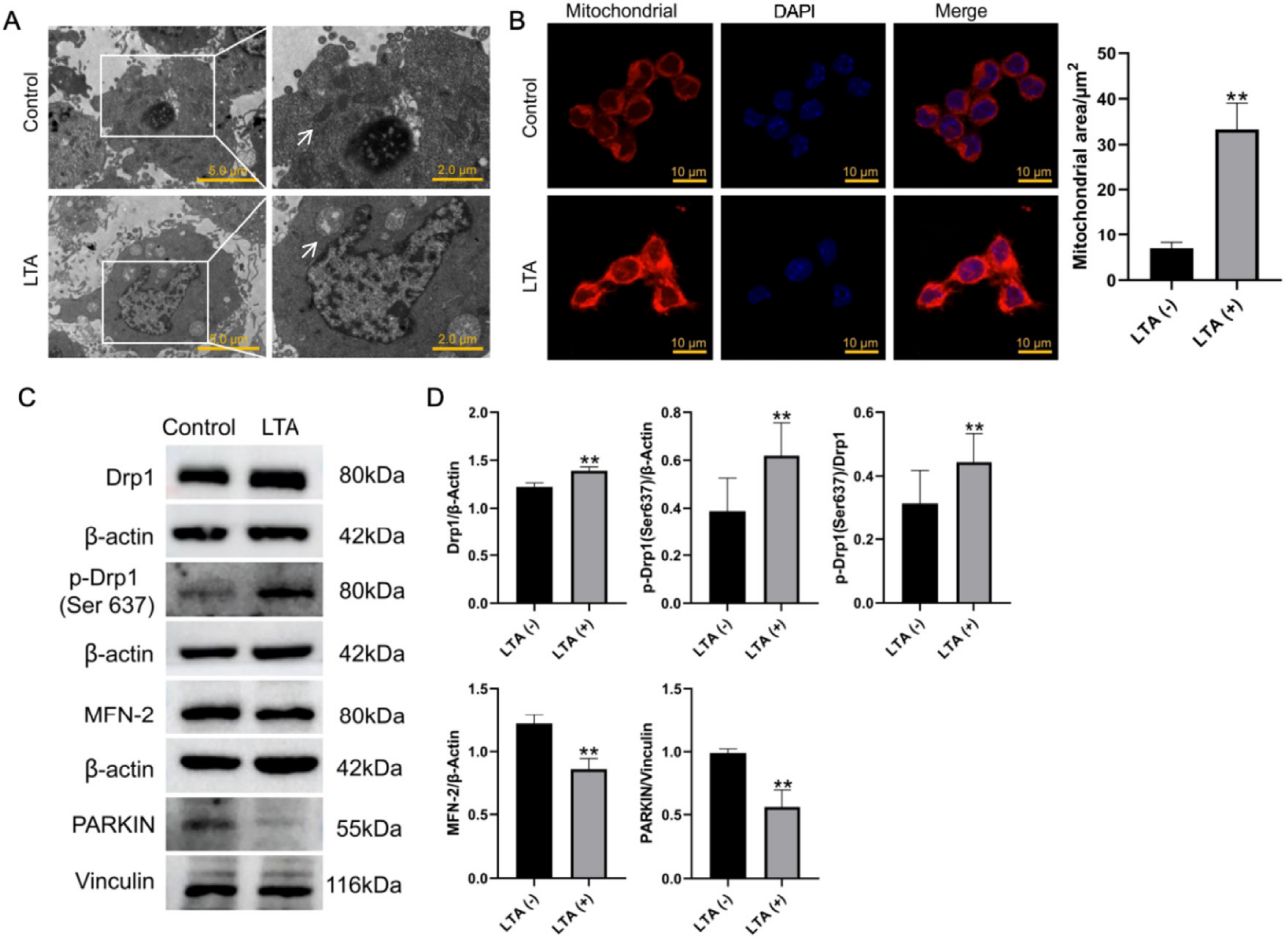
LTA stimulation causes mitochondrial morphological changes. A, TEM analysis of macrophages stimulated with lipoteichoic acid for 36 hours. The right panels are higher-magnification views of the boxed regions in the left panels. White arrows indicate mitochondria. B, Morphological staining and quantitative analysis of mitochondria in macrophages stimulated by lipoteichoic acid for 36 hours (**, *P* < .01). C and D, Expression and quantitative analysis of mitochondrial dynamic proteins and autophagic proteins in macrophages stimulated by lipoteichoic acid for 36 hours (**, *P* < .01).

Given the critical link between mitochondrial morphology and function, we investigated the ultrastructural changes in LTA-stimulated macrophages. As shown in Figure 3A, control cells displayed classic kidney-shaped mitochondria with intact cristae, whereas LTA treatment induced severe pathological swelling, vacuolisation and cristae loss. This damage was quantitatively confirmed by a significant increase in mitochondrial cross-sectional area (Figure 3B). To determine if this structural breakdown resulted from dysregulated dynamics, we analysed key regulatory proteins. Western blot analysis revealed that LTA stimulation elevated levels of total Drp1 and its phosphorylated form (Ser637) while reducing expression of the fusion protein MFN-2 and the autophagy-related protein Parkin (Figure 3C, D). This protein profile indicates an imbalance in mitochondrial dynamics and quality control.

### LTA stimulation causes mitochondrial dysfunction

**Fig. 4:**
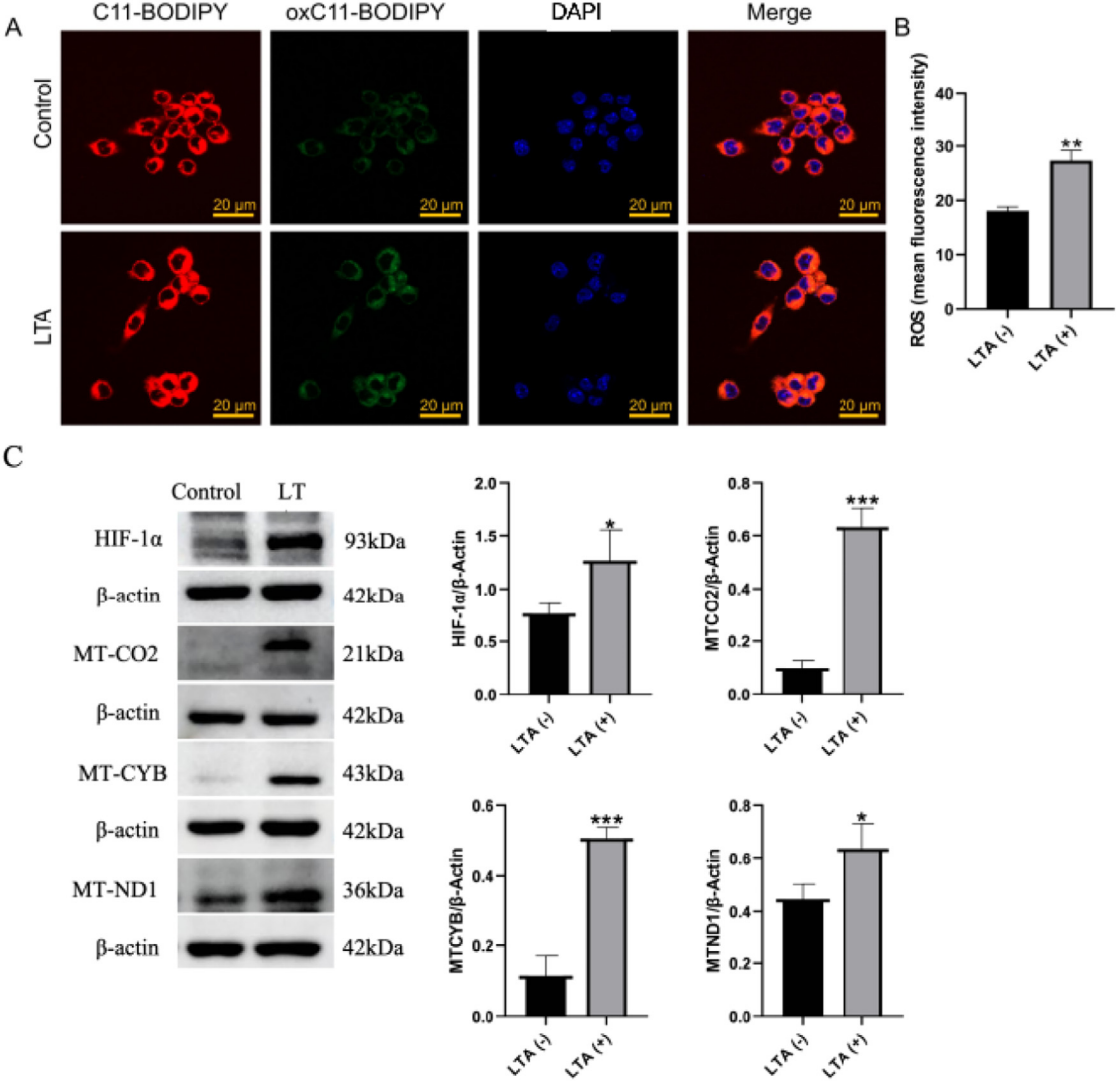
Mitochondrial dysfunction induced by lipoteichoic acid stimulation of macrophages. A, Reactive oxygen species staining of macrophages stimulated by lipoteichoic acid for 36 hours. B, Quantitative analysis of reactive oxygen species in macrophages stimulated by lipoteichoic acid for 36 hours. C, Expression and quantitative analysis of oxidative phosphorylation-related proteins in macrophages stimulated by lipoteichoic acid for 36 hours (*, *P* < .05; ***, *P* < .001).

To assess the impact of LTA on mitochondrial reactive oxygen species (mtROS), we performed MitoTracker Red staining. As shown in Figure 4A, LTA-stimulated cells exhibited a substantial increase in green fluorescence intensity, indicating a significant rise in mtROS levels compared with the control group. At the molecular level, Western blot analysis confirmed the upregulation of the hypoxia-inducible factor HIF-1α and key mitochondrial respiratory chain components (MT-CO2, MT-CYB and MT-ND1). Collectively, these results suggest that LTA stimulation alters mitochondrial oxidative respiration, leading to enhanced mtROS production.

### Mdivi-1 reverses the expression of mitochondrial dynamics proteins

**Fig. 5:**
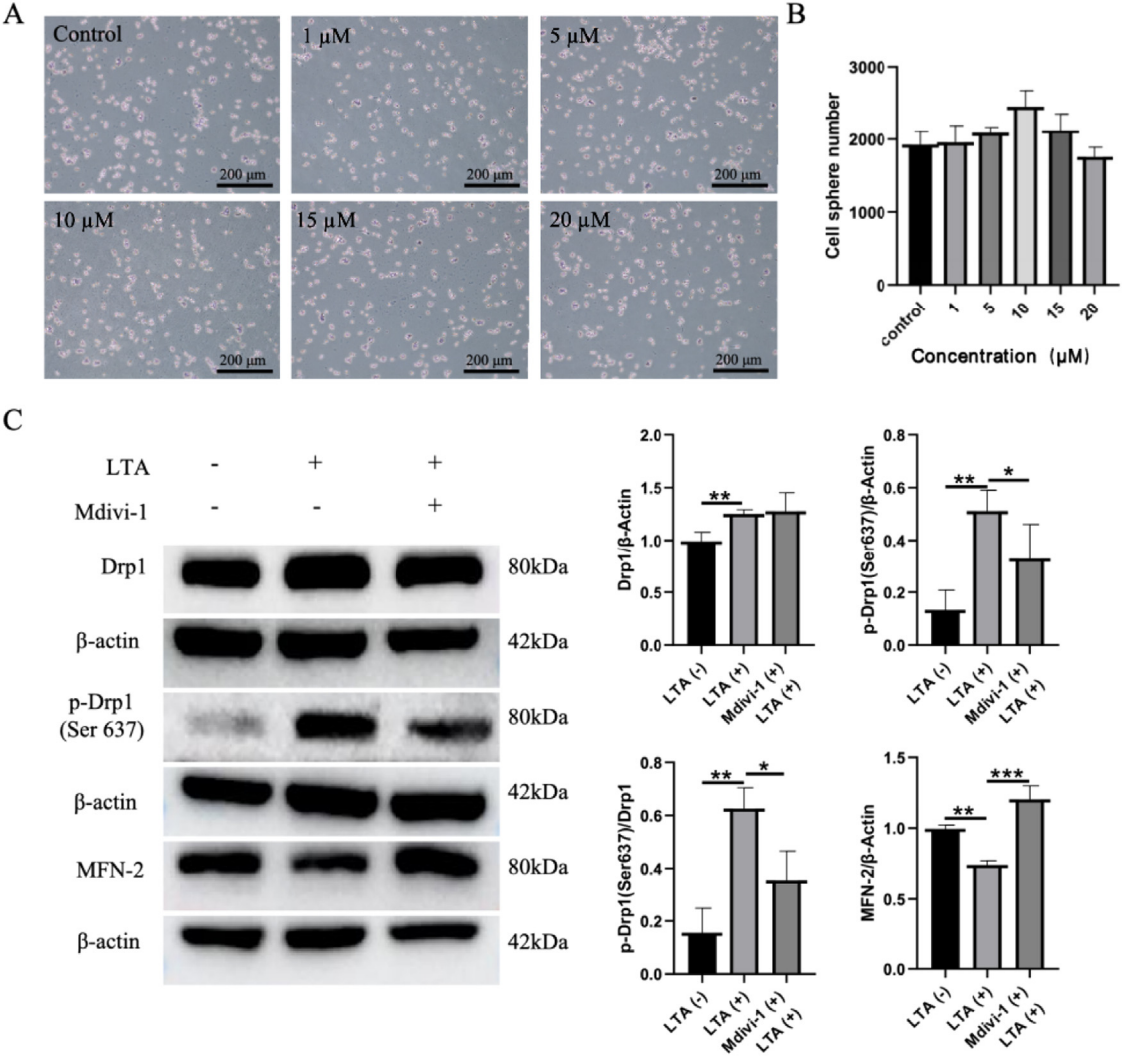
Mdivi-1 reverses mitochondrial kinetic protein expression. A, Cell number of macrophages cultured with different concentrations of Mdivi-1 for 24 hours. B, Quantitative analysis of macrophages cultured with Mdivi-1 at different concentrations for 24 hours. C, Western blot analysis and quantitative analysis of mitochondrial kinetic proteins in RAW264.7 cells induced by Mdivi-1 and lipoteichoic acid.

To investigate the anti-inflammatory potential of Mdivi-1, a 20-µM concentration was selected based on H&E staining, which showed no significant cytotoxicity (Figure 5A, B). At this concentration, Mdivi-1 effectively altered the mitochondrial dynamics profile in macrophages. Western blot analysis showed that it significantly increased the inhibitory phosphorylation of Drp1 (Ser637) and increased MFN-2 expression without affecting total Drp1 levels. This confirmed the successful establishment of our treatment model, characterised by suppressed mitochondrial fission.

### Mdivi-1 restores mitochondrial morphology and function

**Fig. 6:**
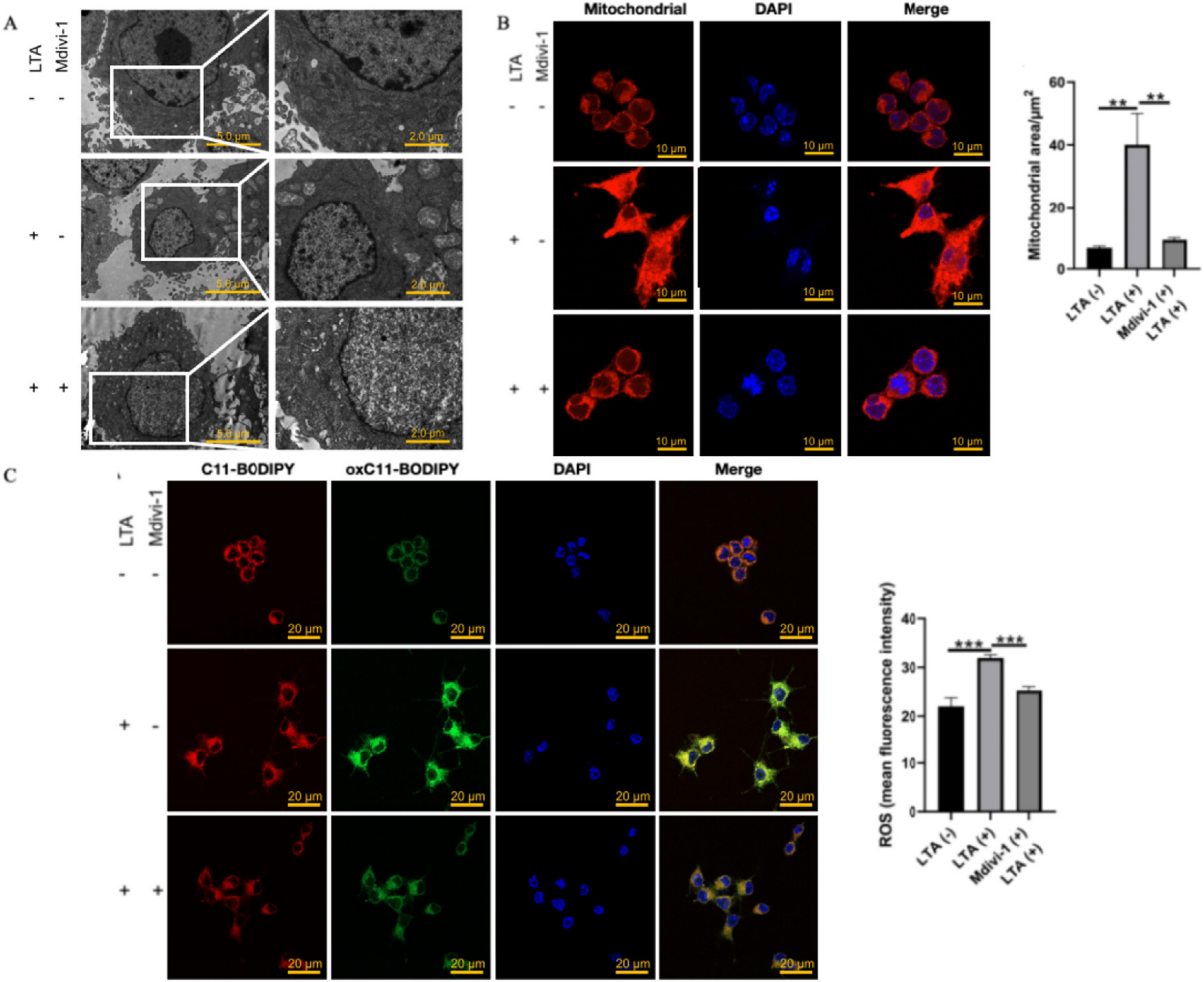
Mdivi-1 treatment of infected macrophages restores mitochondrial morphology and function. A, TEM analysis of macrophage mitochondria. The right panels show higher-magnification views of the boxed regions in the corresponding left panels. B, Morphological analysis of mitochondria in RAW264.7 cells induced by Mdivi-1 and lipoteichoic acid. C, Reactive oxygen species staining analysis of RAW264.7 cells induced by Mdivi-1 and lipoteichoic acid.

To determine the therapeutic effect of Mdivi-1, we analysed its impact on mitochondrial morphology and ROS production. TEM analysis showed that Mdivi-1 treatment largely preserved mitochondrial ultrastructure, with most organelles displaying intact cristae and only minor vacuolation (Figure 6A). Confocal imaging confirmed that mitochondrial morphology in treated cells was restored to a state resembling that of the control group (Figure 6B). Furthermore, mtROS detection indicated a significant decrease in oxidative stress following Mdivi-1 treatment (Figure 6C). These findings collectively demonstrate that Mdivi-1 effectively mitigates LTA-induced mitochondrial damage and dysfunction.

### Mdivi-1 mitigates the inflammatory response in macrophages

**Fig. 7:**
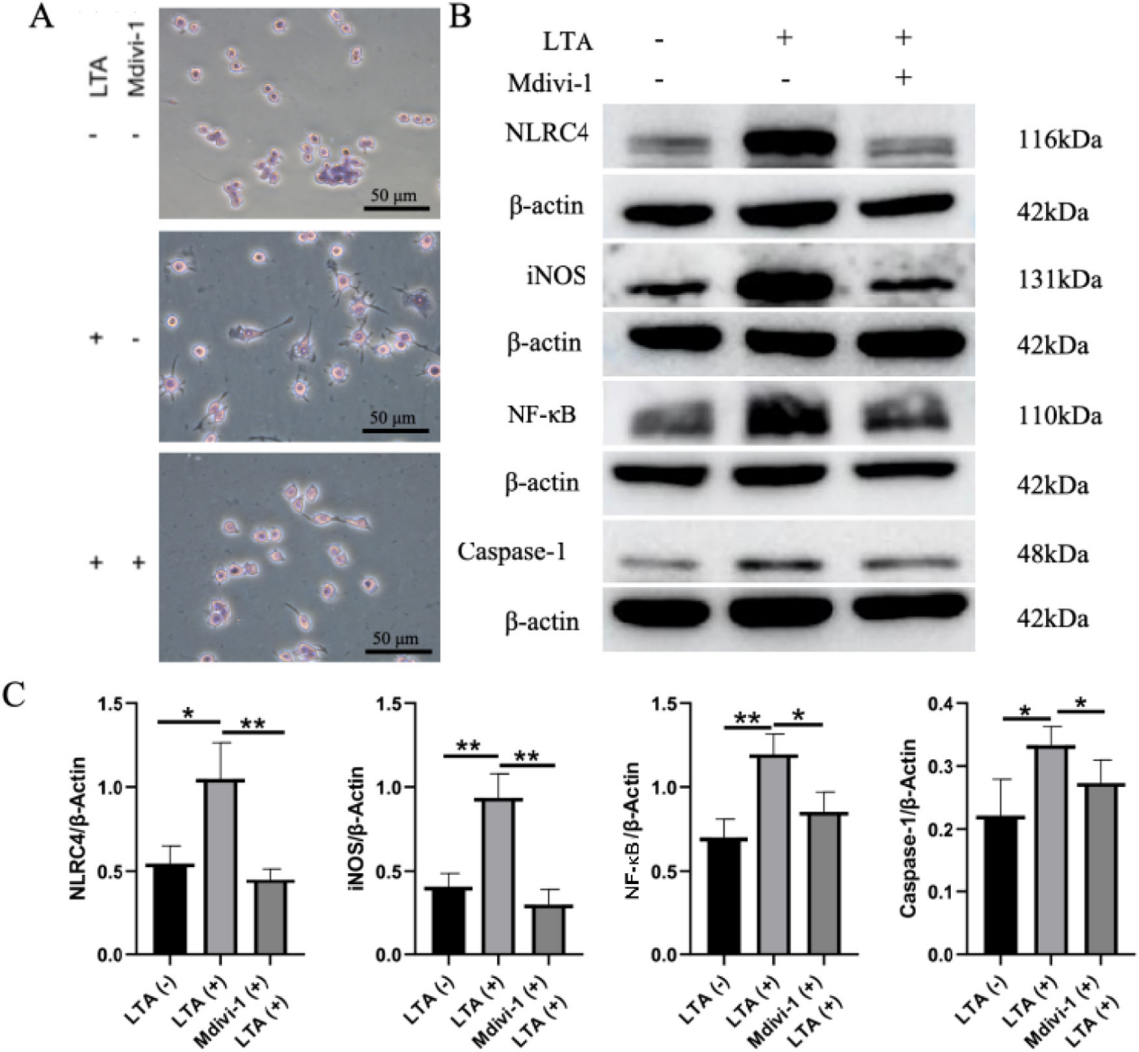
Mdivi-1 reduces the inflammatory response of macrophages. A, H&E staining of RAW264.7 cells induced by Mdivi-1 and lipoteichoic acid. B, Western blot analysis of inflammatory factors in RAW264.7 cells induced by Mdivi-1 and lipoteichoic acid. C, Quantitative analysis of inflammatory factors induced by Mdivi-1 and lipoteichoic acid in RAW264.7 cells (*, *P* < .05; **, *P* < .01).

To evaluate the anti-inflammatory potential of Mdivi-1, we examined its effects on macrophage activation and inflammatory signalling. As shown in Figure 7A, H&E staining revealed a notable reduction in pseudopodia formation in macrophages pre-treated with Mdivi-1 compared to the LTA-only group, indicating suppressed morphological activation. At the molecular level, Western blot analysis demonstrated that Mdivi-1 pre-treatment significantly downregulated key inflammatory mediators, including NLRC4, iNOS, NF-κB and Caspase-1 (Figure 7B, C). These consistent findings at both morphological and molecular levels confirm that Mdivi-1 effectively attenuates the LTA-induced inflammatory response in macrophages.

## Discussion

Most researchers contend that *E. faecalis* is the primary pathogen responsible for refractory apical periodontitis because of its unique ecological adaptations in the post-treatment root canal environment. Clinical evidence demonstrates that *E. faecalis* is the most frequently isolated microorganism in refractory cases, capable of invading dentinal tubules, forming biofilms and surviving under nutrient-deficient conditions.^1^^,^^2^ This clinical relevance validates the use of *E. faecalis* in establishing animal models of RAP because it recapitulates the key pathological features observed in human disease. While animal models provide important pathophysiological insights, cellular models remain essential for mechanistic investigations. Currently, limited literature exists on refractory apical periodontitis, and using *E. faecalis* or its lipoteichoic acid (LTA) to stimulate macrophages or osteoblasts represents a well-established cellular model for investigating this condition.^26^ The selection of LTA in our experimental approach is justified by its documented role as a key virulence factor that elicits pro-inflammatory responses through activation of the NLRP3 inflammasome and promotion of M1 macrophage polarization.^27^^,^^28^ Consequently, this study used LTA-induced macrophages to explore the underlying inflammatory response mechanism, providing a physiologically relevant platform for investigating the mitochondrial aspects of RAP pathogenesis.

To establish a physiologically relevant model, we induced RAP in SD rats by direct inoculation of *E. faecalis* into the mandibular first molar pulp cavity. After a 4-week period, radiographic analysis revealed distinct periapical radiolucencies in infected teeth, and H&E staining demonstrated dense inflammatory cell infiltration within the pulp and periapical tissues. Immunohistochemical exploration further showed significantly elevated iNOS expression concomitant with reduced levels of the mitochondrial proteins MFN-2 and Parkin in the lesion areas. These findings collectively confirm the successful establishment of our RAP animal model and, more importantly, implicate both pro-inflammatory M1 macrophage polarisation and mitochondrial dysfunction in the pathogenesis of *E. faecalis*–induced apical periodontitis. Building on this *in vivo* evidence, we subsequently used an LTA-stimulated macrophage model to mechanistically investigate the underlying inflammatory response.

As depicted in Figure 2, LTA stimulation induced characteristic pseudopod differentiation in macrophages, confirming cellular activation at the morphological level. At the molecular level, western blot analysis demonstrated significantly elevated expression of key inflammatory mediators—NLRC4, Caspase-1, iNOS, and NF-κB. The upregulation of NLRC4 inflammasome components and its effector Caspase-1 provides direct evidence for inflammasome activation, representing the canonical pathway for IL-1β maturation and release.^29^ Concurrently, the coordinated increase in NF-κB activation and iNOS expression confirms a broad pro-inflammatory shift characteristic of M1 macrophage polarisation, a state intrinsically associated with TNF-α production.^30^^,^^31^ This multi-level assessment—from morphological changes to specific molecular pathways—robustly validates the inflammatory phenotype of our model through 2 interconnected axes: inflammasome activation and M1 polarisation, providing a solid foundation for subsequent mechanistic investigations.

Currently, there are no precise reports concerning mitochondrial morphology and function in LTA-infected macrophages. In our study, we observed alterations in mitochondrial morphology and functionality in LTA-induced macrophages and investigated the underlying mechanisms. As demonstrated in Figure 3, using transmission electron microscopy and confocal microscopy, we noted that mitochondria in LTA-induced macrophages became swollen with a loss of internal cristae structure, impacting mitochondrial morphology and structure. This pathological swelling represents a distinct form of mitochondrial damage that directly impacts organelle function. Our investigation revealed that these morphological abnormalities are underpinned by significant dysregulation of mitochondrial dynamics and autophagy. The observed increase in inhibitory phosphorylation at Drp1 Ser637 suggests a compensatory cellular response to restrain excessive fission.^32^ However, the presence of mitochondrial swelling and vacuolisation indicates that this protective mechanism was ultimately overwhelmed. The severity of mitochondrial damage observed in our model strongly implies the concurrent activation of pro-fission pathways, most notably through Drp1 Ser616 phosphorylation, which represents a key mechanism for fission promotion that warrants direct examination in future studies. Mitochondrial health relies on a balance between two processes: fusion to repair damage and fission to remove irreparable components. Our findings reveal that LTA stimulation triggers a vicious cycle of mitochondrial damage and inflammatory signalling. The core of this pathology lies in the simultaneous disruption of both mitochondrial fusion (because of reduced MFN-2) and fission (because of dysregulated Drp1), creating a dual failure in quality control that traps mitochondria in a swollen, dysfunctional state.^33^^,^^34^ This is compounded by impaired clearance, as evidenced by decreased Parkin, which prevents the timely removal of damaged organelles.^35^^,^^36^ Consequently, these dysfunctional mitochondria produce excess ROS, as shown in Figure 4. This elevated ROS acts as a signalling molecule that upregulates HIF-1α, a known driver of M1 pro-inflammatory macrophage polarisation.^37^ The concomitant increase in mitochondrial respiratory proteins (MT-CO2, MT-CYB, MT-ND1) suggests enhanced oxidative respiration as a likely source of this excessive ROS, thereby completing a self-reinforcing cycle of metabolic dysfunction and inflammation.

Mdivi-1, a selective mitochondrial fission inhibitor, exerts broad cytoprotective effects by regulating mitochondrial dynamics, mitophagy, ATP production, immune responses and Ca²⁺ homeostasis.^38^ Accumulating evidence from *in vivo* and *in vitro* studies supports its therapeutic potential. For instance, Su et al. demonstrated that Mdivi-1 attenuates atherosclerotic plaque formation by suppressing aberrant mitochondrial dynamics and ROS overproduction in ApoE⁻/⁻ mice and ox-LDL-stimulated macrophages.^39^ Similarly, Zhang et al. reported that Mdivi-1 alleviates neurological and synaptic dysfunction in a mouse model,^40^ whereas Deng et al. showed its efficacy in ameliorating acute lung injury via modulation of pathological mitochondrial fission in LPS-induced macrophages.^41^ However, no studies have yet reported on the effects of Mdivi-1 in apical periodontitis or LTA-induced macrophage models. A pivotal recent study by Yang et al. has further established the therapeutic role of Mdivi-1 in apical periodontitis, demonstrating its ability to alleviate bone erosion by inhibiting Drp1-mediated mitochondrial fission and NLRP3-dependent M1 macrophage polarisation in a Pg-LPS model.^42^

Whereas this foundational work confirms the importance of the Drp1 pathway in periapical disease, our study unveils a distinct and more complex mechanistic narrative within LTA-induced macrophages. Our data reveal that Mdivi-1 not only directly inhibits Drp1 but also triggers a profound rewiring of the mitochondrial dynamics network, as evidenced by the reduction in p-Drp1 (Ser637) and restoration of MFN-2. We propose a model wherein the primary inhibition of Drp1 GTPase activity initiates a therapeutic feedback loop. By halting pathological fission, Mdivi-1 alleviates the mitochondrial membrane potential collapse and oxidative stress that characterise the inflammatory state.^19^ This improved mitochondrial fitness then signals back to the cell, rendering the sustained high level of compensatory inhibitory phosphorylation at Ser637 unnecessary, leading to its downregulation. Concurrently, this healthier metabolic milieu, potentially through reduced ROS-mediated suppression and activation of pro-fusion signalling pathways, favours the transcriptional and/or post-translational upregulation of MFN-2. Therefore, Mdivi-1 functions not merely as an inhibitor but as a network rebalancer, which, through its primary action, corrects both the phospho-regulatory landscape and the proteomic composition of the mitochondrial dynamics machinery to restore homeostasis.

In summary, the macrophage model induced by LTA demonstrates that macrophages differentiate pseudopods and exhibit increased expression of inflammatory factors such as NLRC4, iNOS, NF-κB and Caspase-1, indicating a pronounced inflammatory response. Further investigations have revealed abnormalities in mitochondrial morphology and structure, along with significant alterations in mitochondrial dynamics and autophagy-related proteins, notably p-Drp1(ser637), MFN-2 and Parkin. Concurrently, there is an increase in the expression of reactive oxygen species and the respiratory oxidative proteins HIF-1α, MT-CO2, MT-CYB and MT-ND1. The application of Mdivi-1 modifies mitochondrial dynamics proteins, enhances mitochondrial morphology and functionality and mitigates the inflammatory response. This study not only advances our understanding of the pathogenesis of refractory apical periodontitis but also positions Mdivi-1 as a partially evidence-based treatment for this condition.

Although this study elucidates a novel mechanism linking mitochondrial dynamics to macrophage polarisation in the pathogenesis of refractory apical periodontitis, several limitations must be acknowledged. The reliance on a macrophage cell line and a single bacterial component (LTA) inevitably simplifies the complex polymicrobial and tissue microenvironment of clinical apical periodontitis. To address these limitations and validate the therapeutic potential of Mdivi-1, we propose a hierarchical research pathway. First, *in vitro* models should be advanced to incorporate primary human macrophages and multispecies biofilms to better approximate disease complexity. Second, and most critically, the efficacy of Mdivi-1 must be evaluated in a physiologically relevant *in vivo* context. We plan to use a rat model of *E. faecalis*–induced apical periodontitis, employing local administration of Mdivi-1 via a controlled-release system. Therapeutic outcomes will be rigorously assessed through micro-CT quantification of bone loss and immunohistochemical analysis of mitochondrial and immunomodulatory dynamics within the lesions. These investigations are designed to explore the translational path of Mdivi-1 from a mechanistic tool to a therapeutic candidate by connecting *in vitro* findings with *in vivo* function. This work is intended to inform future strategies for immunometabolic intervention in endodontic disease.

## Conclusion

Animal studies have demonstrated high expression of pro-inflammatory macrophages and abnormal mitochondrial-related protein levels in *E. faecalis*–induced apical periodontitis lesions. *In vitro* experiments further revealed mitochondrial morphological and functional disruptions in lipoteichoic acid–stimulated macrophages, which were ameliorated by the mitochondrial division inhibitor Mdivi-1. Therefore, the application of Mdivi-1 may enhance the anti-inflammatory capacity of macrophages by restoring mitochondrial structure and function, offering a novel therapeutic strategy for the treatment of apical periodontitis.

## Clinical significance

This study highlights the potential of targeting mitochondrial dysfunction as a therapeutic strategy for refractory apical periodontitis. By restoring mitochondrial dynamics and function with Mdivi-1, macrophage-mediated inflammation can be mitigated, thereby enhancing tissue healing and resolving inflammation. These findings provide a foundation for developing novel immunomodulatory treatments aimed at improving clinical outcomes in patients with persistent apical periodontitis.

## Author contributions

*Conceptualisation:* Shuang Pan, Weiwei Zhang

*Supervision:* Shuang Pan, Weiwei Zhang

*Design:* Shuang Pan, Weiwei Zhang, Siyao Liu, Xin Liu

*Experiments:* Siyao Liu, Xin Liu

*Formal analysis:* Guixin Li

*Writing—initial draft:* Siyao Liu, Xin Liu

*Writing—revision and editing:* Huan Liu

## Conflict of interests

None declared
